# Chemotherapy of HER2- and MDM2-Enriched Breast Cancer Subtypes Induces Homologous Recombination DNA Repair and Chemoresistance

**DOI:** 10.3390/cancers13184501

**Published:** 2021-09-07

**Authors:** Marcin Herok, Bartosz Wawrzynow, Marta J. Maluszek, Maciej B. Olszewski, Alicja Zylicz, Maciej Zylicz

**Affiliations:** 1 International Institute of Molecular and Cell Biology in Warsaw, 02-109 Warsaw, Poland; mherok@iimcb.gov.pl (M.H.); bartosz.wawrzynow@fnpventures.com (B.W.); mmaluszek@iimcb.gov.pl (M.J.M.); maciej@olszewski.science (M.B.O.); alicja.zylicz@iimcb.gov.pl (A.Z.); 2 Nencki Institute of Experimental Biology, Polish Academy of Sciences, 02-093 Warsaw, Poland; 3 Institute of Biochemistry and Biophysics, Polish Academy of Sciences, 02-106 Warsaw, Poland; 4 Center for Translational Medicine, Warsaw University of Life Sciences, 02-787 Warsaw, Poland

**Keywords:** DNA damage repair, homologous recombination, MDM2, NBN, chemoresistance

## Abstract

**Simple Summary:**

MDM2 is a protein responsible for negative regulation of the p53 tumor suppressor. In addition, MDM2 exhibits chaperone-like properties similar to the HSP90 molecular chaperone. Multiple studies revealed that MDM2 is deeply involved in cancer development and progression. Some recently published results indicate that the role of MDM2 in DNA repair inhibition is more complex than previously thought. We show that MDM2 is directly involved in the homologous recombination DNA repair, and its chaperone-like activity is crucial for this function. The DNA repair inhibition is a result of inefficient MDM2 dissociation from the NBN protein complex. When cancer cells are treated with chemotherapy, MDM2 can be easily released from the interaction and degraded, resulting in effective homologous recombination DNA repair, which translates into the acquisition of a chemoresistant phenotype by the tumor. This knowledge may allow for identification of the patients that are at particular risk of tumor chemoresistance.

**Abstract:**

Analyzing the TCGA breast cancer database, we discovered that patients with the HER2 cancer subtype and overexpression of MDM2 exhibited decreased post-treatment survival. Inhibition of *MDM2* expression in the SKBR3 cell line (HER2 subtype) diminished the survival of cancer cells treated with doxorubicin, etoposide, and camptothecin. Moreover, we demonstrated that inhibition of *MDM2* expression diminished DNA repair by homologous recombination (HR) and sensitized SKBR3 cells to a PARP inhibitor, olaparib. In H1299 (*TP53*^−/−^) cells treated with neocarzinostatin (NCS), overexpression of MDM2 WT or E3-dead MDM2 C478S variant stimulated the NCS-dependent phosphorylation of ATM, NBN, and BRCA1, proteins involved in HR DNA repair. However, overexpression of chaperone-dead MDM2 K454A variant diminished phosphorylation of these proteins as well as the HR DNA repair. Moreover, we demonstrated that, upon NCS treatment, MDM2 K454A interacted with NBN more efficiently than MDM2 WT and that MDM2 WT was degraded more efficiently than MDM2 K454A. Using a proliferation assay, we showed that overexpression of MDM2 WT, but not MDM2 K454A, led to acquisition of resistance to NCS. The presented results indicate that, following chemotherapy, MDM2 WT was released from MDM2-NBN complex and efficiently degraded, hence allowing extensive HR DNA repair leading to the acquisition of chemoresistance by cancer cells.

## 1. Introduction

DNA damage in the form of a single-strand break (SSB) or a double-strand break (DSB) induces activation of the DNA damage response (DDR) signaling pathway [1]. The more toxic DSB can be repaired by homologous recombination (HR) or non-homologous end-joining (NHEJ) DNA repair pathways. In rapidly replicating cancer cells, DSB are created at replication forks or by chemotherapeutic agents. They are repaired by the HR DNA repair mechanism, which uses the undamaged sister chromatid as a repair template [2], predominantly during the S, G_2_, and M phases of the cell cycle [3,4]. The NHEJ DNA repair pathway does not rely on undamaged DNA molecules, is much more error-prone, and operates predominantly in the G_1_ phase [5,6]. Very efficient repair of DSB in cancer cells could lead to the inhibition of apoptosis and the acquisition of chemoresistance [7]. Both HR and NHEJ pathways are initiated by positioning of the MRN complex (MRE11/RAD50/NBN) on DSB [5,6]. After phosphorylation of histone H2AX, the MRN complex positioned on DSB recruits inactive Ataxia-telangiectasia mutated (ATM) kinase dimers and activates them [8]. Activated ATM intensifies the DNA damage response by further phosphorylation of H2AX histones and other downstream components of this pathway: checkpoint kinases 1 and 2 (CHK1 and CHK2), breast cancer 1 (BRCA1), and p53, as well as components of the MRN complex. These events trigger RAD51-dependent, homology-directed DNA repair [9]. 

Mouse double minute 2 (MDM2) is a negative regulator of the p53 tumor suppressor protein. Under normal, non-stress conditions, MDM2 inhibits the transcriptional activity of p53 and is responsible for p53 ubiquitination, targeting it for proteasomal degradation [10,11]. Consistently, in healthy adult breast duct cells, the major tumor suppressor p53 is not detectable, while MDM2 and MDM4 are observed at high levels [12,13]. In response to cellular stress, including genotoxic stress, p53 is stabilized by the disruption of MDM2–p53 interaction and can act as a transcription factor leading to tumor suppression [11,14,15]. p53 induces or attenuates expression of hundreds of genes, including *MDM2* and genes responsible for apoptosis, cell cycle arrest, the DNA damage response (DDR), autophagy, and DNA repair [16]. The *TP53* gene is often mutated in cancer cells [17]. Inactivation of p53 provides cancer cells a growth and survival advantage. The majority of mutations in *TP53* gene are missense mutations [17]. The resulting p53 protein variants with a single amino acid substitution can acquire oncogenic properties [18]. MDM2 not only shows E3 ligase activity [19] and ATP-dependent molecular chaperone-like activity [20] but is also involved in the transrepression of other genes [21,22], translational control [23,24], and the maintenance of genome stability [25]. Some of these MDM2 activities are independent of p53 [26]. Elevated levels of MDM2 protein strongly correlate with an increased risk of cancer [13,27,28]. Furthermore, MDM2 overexpression was demonstrated in a variety of tumors [27,28,29,30,31,32,33,34,35,36,37,38,39]. In human breast cancer, the MDM2 protein level was identified as a prognostic biomarker [40]. Overexpression of *MDM2* gene leads to a growth/survival advantage for cancer cells not only by p53 inhibition but also by induction of p53-independent pro-survival mechanisms, including inhibition of tumor suppressor activity of retinoblastoma (Rb) [41] or (E2F transcription factor 1) (E2F1) [42]. Investigating p53-independent activity of MDM2, Eischen laboratory identified three members of the MRN complex (namely, MRE11, RAD50, and NBN), which co-immunoprecipitated with MDM2 from HeLa cells lysate [43]. The direct and specific interaction between MDM2 and NBN led to the delay or even the inhibition of γH2AX phosphorylation and DNA break repair, as measured using comet assay [25,43,44]. Elevated levels of MDM2 in the absence of p53 resulted in chromatid and chromosome breaks. MDM2-dependent delay of DNA repair resulted in an increased transformation potential of p53-null fibroblasts [44]. The involvement of MDM2 in the inhibition of DNA repair is well documented, but several findings have recently been published that contradict previous statements. Cancer cells with *MDM2* amplification are selectively resistant to treatment with drugs introducing DSB (inhibitors of topoisomerase II). These tumor cells have reduced DDR after treatment with doxorubicin or etoposide [45]. Moreover, MDM2 inhibitor (nutlin-3) delayed DNA repair of DSB in pancreatic ductal adenocarcinoma (PDAC model), suggesting that MDM2 inhibitors block MDM2 activities needed for effective DNA repair [46]. MDM2 inhibitors synergize with topoisomerase II inhibitors to induce p53-independent pancreatic cancer cell death [46]. 

Herein, to answer the question of how *MDM2* overexpression may simultaneously promote genome instability and the survival of cancer cells treated with chemotherapeutics, we verified the hypothesis that in rapidly replicating cancer cells high levels of MDM2 protein inhibit HR-based DSB DNA repair by interaction with the MRN complex. We demonstrated that, following treatment with chemotherapeutics, MDM2 is released from the MRN complex in an ATP-dependent reaction, triggering efficient homologous recombination DNA repair, thereby allowing cancer cells to survive the exposure to DNA-damaging drugs.

## 2. Materials and Methods

### 2.1. Breast Cancer Patients Survival Analysis

Breast cancer patients’ clinical and genetic data were retrieved from The Cancer Genome Atlas (TCGA) using the UCSC Xena browser (http://xena.ucsc.edu, accessed on 1 December 2019). After proper filtering (Study: “TCGA Breast Cancer (BRCA),” Phenotype: “Primary Tumor,” “PAM50 subtype from RNAseq data (TCGA AWG),” “OS.time,” “vital status,” Genomic: “Somatic Mutation: TP53,” “Gene Expression: MDM2”) the data of 807 patients were used for survival analysis. Samples from specific molecular subtypes of breast cancer were divided into two groups (MDM2 high and low) by the median value of *MDM2* gene expression in that subtype. The statistical significance of differences in survival was assessed by a log-rank test (Mantel–Haenszel) using GraphPad Prism 6 software.

### 2.2. Cell Culture, Transfection, and Treatment 

All cell lines: MCF7 (HTB-22), SKBR3 (HTB-30), MDA-MB-231 (HTB-26), MDA-MB-468 (HTB-132), and H1299 (CRL-5803) were purchased from the American Type Culture Collection (ATCC, Manassas, United States) and cultivated according to the supplier’s documentation. Transient transfection with siRNA was performed with GenMute siRNA Transfection Reagent (SignaGen, Frederick, MD, USA), according to the manufacturer’s manual, using 40 nM of specific siRNA (Thermo Fisher Scientific, Waltham, MA, USA): control—(AM4613), MDM2–siMDM2#1 (s8630), siMDM2#2 (AM51334), BRCA1–siBRCA1 (s459), and RAD51–siRAD51 (VHS40453). Cells were seeded on 10-cm-diameter culture dishes (2,400,000 cells/plate) 24 h before first transfection. Transfection was performed twice, 24 h apart. Gene silencing efficiency was verified with RT-PCR and western blot analysis. Transient transfection with plasmids was performed with Lipofectamine 2000 Transfection Reagent (Thermo Fisher Scientific), according to the manufacturer’s manual. Cells were treated with indicated concentrations of doxorubicin, camptothecin, etoposide, olaparib (all Selleckchem, Houston, TX, USA), and neocarzinostatin (Sigma-Aldrich, St. Louis, MO, USA). 

### 2.3. Viability Assay

Metabolic activity (viability) was measured with resazurin reduction assay. Cells were seeded on 96-well plates (12,800 cells/well), and, following drug treatment, the plates were rinsed with PBS. Resazurin (15 μg/mL, R7017, Sigma-Aldrich) in a standard culture medium was added. Plates were incubated for 4 h (37 °C, 5% CO_2_, humidified incubator), and fluorescence was measured at 590 nm (excitation at 560 nm) with Tecan Infinite M1000 plate reader (Tecan, Männedorf, Switzerland). Cell viability is expressed as a percentage of viable treated cells relative to the corresponding control cells. 

### 2.4. Real-Time Cell Proliferation Monitoring with the xCelligence SYSTEM

Real-time cell proliferation monitoring was performed with the RTCA DP xCelligence system (ACEA Biosciences, San Diego, CA, USA). The equipment was placed in a cell culture incubator (37 °C, 5% CO_2,_ humidified incubator). The resistance values measured by the device were quantified as the cell index (CI). Twelve thousand cells were seeded per well of the 16-well E-plates with an area of 0.3 cm^2^ (12,000 cells/well) according to the manufacturer’s procedure. Twenty-four hours after the start of the measurements, the chemotherapeutic agents were added at the appropriate concentrations. Cell proliferation was monitored for the period of time indicated in the respective figures. Data analysis was carried out with the RTCA Software 2.0 (ACEA Biosciences, San Diego, CA, USA) and GraphPad Prism 6.

### 2.5. In-Cell Western

The in-cell western (ICW) Assay is a quantitative immunofluorescence assay performed in 96-well plate format, functionally equivalent to western blot but providing high-throughput capability. Cells were seeded and cultured on 96-well plates (12,800 cells/well). After the treatment, plates were rinsed with PBS and fixed with 100% methanol for 20 min in −20 °C and blocked for 2 h in: 2% BSA, 2% donkey serum, and 0.1% Triton X-100, PBS. The same buffer was used for overnight incubation with primary antibody (1:5000, phospho-H2AX antibody NB100-384, Novus Biologicals). Subsequent steps of the assay were performed according to LI-COR’s in-cell western workflow. The fluorescence signal was measured with Odyssey CLx (LI-COR, Lincoln, NE, USA), and total fluorescence from the antibody was normalized to the counterstain signal corresponding to the number of cells in a well.

### 2.6. Proximity Ligation Assay (PLA)

Cells (76,000 cells/well) were seeded on sterile 12-mm coverslips (P231.1, ROTH), which were placed individually in the wells of a 24-well plate (BD, Franklin Lakes, NJ, USA). After 24 h, cells were washed twice with PBS and fixed with frozen (−20 °C) 100% methanol for 20 min at −20 °C then washed with PBS buffer. The experiments were performed with the use of Duolink In Situ Detection Reagents Red ligation kit (DUO92008, Sigma-Aldrich), Duolink In Situ PLA Probe Anti-Mouse MINUS secondary antibodies (DUO92004, Sigma-Aldrich, St. Louis, MO, USA), and Duolink In Situ PLA Probe Anti-Rabbit PLUS (DUO92002, Sigma-Aldrich, St. Louis, MO, USA). The entire procedure was performed according to the manufacturer’s instructions, using only the reagents provided in the kit. Pictures were taken with confocal fluorescence microscope ZEISS LSM 800 (Oberkochen, Germany).

### 2.7. Co-Immunoprecipitation (Co-IP)

Material for immunoprecipitation was collected from 10-cm plates of fully confluent cells. Cells were lysed in the following IP buffer: 50 mM Tris-HCl pH 8.0; 150 mM NaCl; 0.5% NP-40; 2 mM MgCl_2_; 1 mM EDTA; 10 mM NaF; 1 mM Na_3_VO_4_-2H_2_O. Cells were lysed for 30 min on ice then sonicated (2 × 5 s, 25% power) and centrifuged for 10 min at 12,000 rpm, 4 °C. The volume of lysate containing a total of 2 mg of protein was transferred to a new tube and diluted with IP buffer to a concentration of approx. 10 μg/μL. Five μL of Dynabeads Protein G (10004, Thermo Fisher Scientific, Waltham, MA, USA) pre-washed in IP buffer was added and incubated for 30 min at 4 °C with agitation. This step was to eliminate non-specific binding of the lysate components to the surface of the magnetic beads. In parallel, capture antibodies (2 µg) were added to the lysate and incubated overnight (ca. 16 h) at 4 °C with rocking. Ten µL of pre-blocked Dynabeads Protein G was then washed in IP buffer and added to each sample. The sample was incubated for 60 min at 4 °C with rocking. The magnetic beads were rinsed three times with cold IP buffer for 5 min at 4 °C with rocking. Finally, the beads were resuspended in 10 µL of RIPA buffer and heated at 98 °C for 10 min prior to performing SDS-PAGE.

### 2.8. In-Vivo Protein Ubiquitination Assay

H1299 cells were seeded in a 6-well dish at a density of 280,000 cells/well. The next day, cells were transfected with 2 µg of plasmid encoding the HA-tagged ubiquitin and 2 µg of plasmid encoding the MDM2 WT or MDM2 K454A gene using Lipofectamine 2000 (Life Technologies, Carlsbad, CA, USA) according to the manufacturer’s protocol. Six hours after transfection, the transfection medium was replaced with fresh culture medium. Twenty-four hours after transfection, 150 nM neocarzinostatin (NCS; Sigma-Aldrich, St. Louis, MO, USA) and 20 nM proteasome inhibitor MG132 (Tocris, Bristol, UK) were added to the medium for 30 min. After that time, NCS was removed, and incubation with MG132 continued for an additional 3.5 h. Cells were then washed with cold PBS and lysed in 300 μL of RIPA buffer directly in a dish placed on ice. The samples were centrifuged (10 min, 13,000 rpm, 4 °C), the supernatants were collected, and protein concentration was measured by the Bradford method. Lysate samples with a total protein content of 40 µg each were saved as IP input controls. Protein G-bound agarose beads were coated with the antibody: 3.5 µg of anti-MDM2 (4B2) antibody was added to 10 µL of RIPA pre-washed 50% agarose beads and incubated for 2 h. Antibody-coated agarose beads were added to the total cell lysates and incubated at 4 °C for 16 h. The beads were then washed four times for 5 min in RIPA buffer. For protein denaturation, the beads were heated at 98 °C for 10 min in RIPA with Laemmli buffer. The samples were centrifuged, and the supernatants were collected. All samples were separated on a 4–20% gradient polyacrylamide gel for western blot analysis. The MDM2 protein in control samples (input) was detected with 4B2 antibody, and ubiquitinated MDM2 protein was detected with anti-HA antibody (Y11, Santa Cruz Biotechnology, Dallas, TX, USA).

### 2.9. Antibodies and Primers

The following antibodies were used for western blot and co-immunoprecipitation: p53 (DO-1, 1:10,000) and MDM2 (4B2, 1:250, SMP14, 1:250) were a kind gift from B.Vojtesek (Moravian-Biotechnology Ltd., Brno, Czech Republic ), GAPDH (#2118, Cell Signaling, 1:5000), β-actin-HRP (AC-15, Sigma-Aldrich, St. Louis, MO, USA, 1:10,000), NBN (NB100-143, Novus Biologicals, Littleton, CO, USA, 1:5000), pNBN (Ser343) (ab47272, Abcam, Cambridge, UK, 1:250), MRE11 (NB-473, Novus Biologicals, Littleton, CO, USA, 1:1000), RAD50 (NB100-154, Novus Biologicals, Littleton, CO, USA, 1:5000), pATM (Ser1981) (200-301-400, Rockland, Gilbertsville, PA, USA, 1:500), pBRCA1 (Ser1524) (#9009, Cell Signaling, Danvers, MA, USA, 1:250), Chk2 (ab47433, Abcam, Cambridge, UK, 1:500), and pChk2 (Thr68) (#2197, Cell Signaling, Danvers, MA, USA, 1:250). Secondary antibodies: anti-Mouse HRP (A9917, Sigma-Aldrich, St. Louis, MO, USA, 1:10,000), anti-Rabbit HRP (Sigma-Aldrich, St. Louis, MO, USA, A0545, 1:10,000), anti-Mouse IRDye^®^ 800CW (926-32212, LI-COR, Lincoln, NE, USA, 1:15,000), and anti-Rabbit IRDye^®^ 680LT (926-68023, LI-COR, Lincoln, NE, USA, 1:15,000). The following primers were used for RT-PCR: MDM2 F: GGAGATTTGTTTGGCGTGC, MDM2 R: AGTCCGATGATTCCTGCTGA, GAPDH F: AAGGTGAAGGTCGGAGTCAA, GAPDH R: TGAGGTCAATGAAGGGGTCA, BRCA1 F: GATTTATCTGCTCTTCGCGT, BRCA1 R: AGGTTCCTTGATCAACTCCA, RAD51 F: GCTGCGGACCGAGTAA, and RAD51 R: TTCTTCACATCGTTGGCATT. RT-PCR conditions: initial denaturation: 95 °C, 10 min; 34 cycles: denaturation: 95 °C, 15 s; annealing: 56 °C, 30 s; extension: 72 °C, 30 s. Equipment used: LightCycler 96 Real-Time PCR System (Roche, Basel, Switzerland).

### 2.10. Homologous Recombination Assay Kit

Homologous Recombination Assay Kit (#35600, Norgen Biotek, Thorold, ON, Canada) is a sensitive tool for measuring the efficiency of homologous recombination through RT-PCR. Cells were seeded on 12-well plates (152,000 cells/well) and transfected with plasmid mix (dl-1, dl-2, 300 ng) or the control plasmid (300 ng per well). After 24 h, cellular DNA was isolated using the Cell and Tissue DNA Isolation Kit (#53100, Norgen Biotek, Thorold, ON, Canada), according to the manufacturer’s protocol. One hundred ng of DNA was used as a template for RT-PCR (SG qPCR Master Mix, EURX, Gdansk, Poland) with primers supplied in the kit. The results were analyzed using LightCycler 96 Real-Time PCR System (Roche, Basel, Switzerland) software. 

### 2.11. Homologous Recombination GFP Reporter Assay

H1299 cells, stably transfected with GFP/GFP* reporter (Dr. Slabicki gift), were seeded on 12-well plates (152,000 cells/well) and, after 24 h, transfected with plasmid encoding I-SceI endonuclease (300 ng per well). Forty-hour post-transfection cells were collected, resuspended in 1 mL of PBS, and placed on ice for flow cytometry analysis. Correct repair of the GFP/GFP* reporter by homologous recombination leads to the creation of a functional *GFP* gene whose expression can be observed as cellular fluorescence. HR repair efficiency was calculated as the percentage of GFP-positive cells in all living cells.

### 2.12. Statistical Analysis

The experimental data were processed in Microsoft Excel and then analyzed in GraphPad Prism 6. One-way or two-way analysis of variance (ANOVA) was used to compare the tested variants with control samples, depending on the experiment design, followed by Tukey’s honest significance test. All experiments were performed with at least three independent biological replicates. Appropriate controls and technical repetitions were used depending on the type of experiment. Statistical significance: * *p* < 0.05, ** *p* < 0.01, *** *p* < 0.001. Error bars represent SD.

## 3. Results

### 3.1. An Elevated Level of MDM2 Transcript Correlates with a Worse Prognosis for Survival in Patients with an HER2-Enriched Molecular Subtype of Breast Cancer

**Figure 1:**
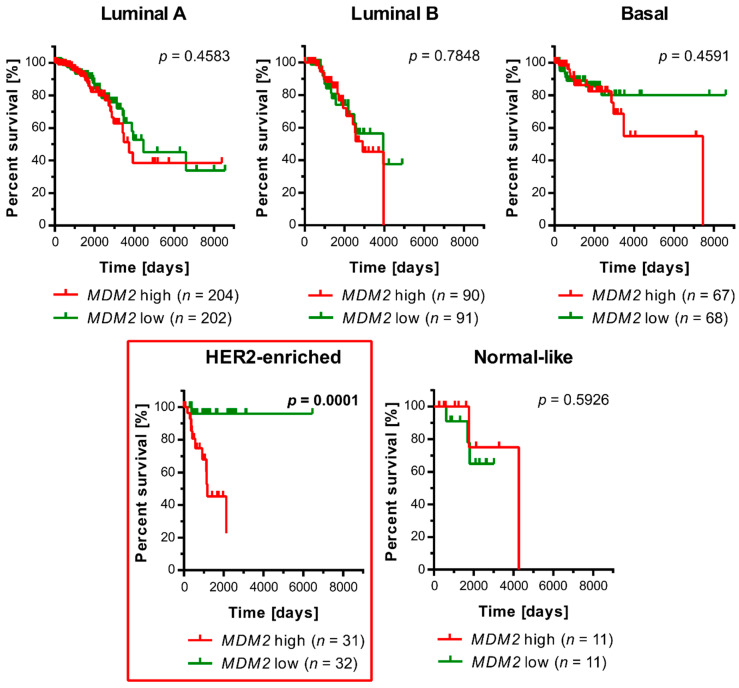
Kaplan–Meier survival curves for overall survival of TCGA breast cancer patients divided by molecular subtypes of breast cancer with high or low *MDM2* gene expression. The log-rank test showed a statistically significant difference between the patient survival curves for the HER2-enriched subtype (red box). Patients were assigned to groups with high or low *MDM2* transcript levels based on the median value specific to each subtype.

Previously, we and others demonstrated that MDM2 overexpression in non-small cell lung cancer [28] and breast cancer [39] decreased overall patient survival after treatment. We used clinical data presented in (the Cancer Genome Atlas Program) TCGA to extend this research and investigate if a correlation between overexpression of *MDM2* gene and a decrease in patient survival is found in any of the molecular subtypes of breast cancer. As shown in Figure 1, in the HER2-enriched molecular subtype, where 65% of the patients bear mutations in *TP53* (Figure S1), there was a very strong association (*p* = 0.0001; Figure 1) between *MDM2* overexpression and a decrease in overall survival.

### 3.2. Downregulation of MDM2 Gene Expression Leads to Decreased Survival of SKBR3 Cells (HER2-Enriched Subtype) after Treatment with DNA-Damaging Chemotherapeutic Agents

**Figure 2:**
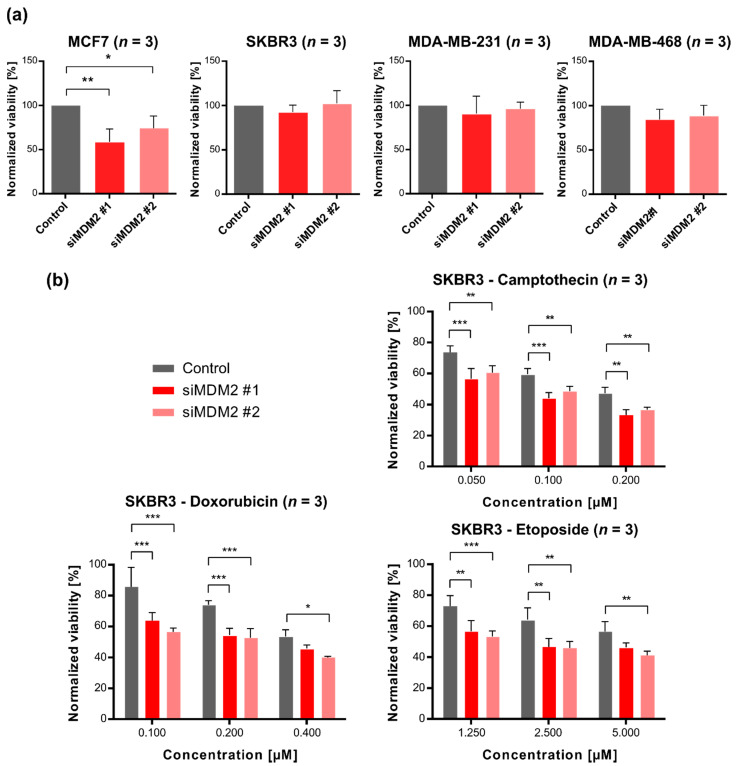
Cell viability after silencing of *MDM2* gene expression with and without treatment with chemotherapeutics. Cell survival was assessed by metabolic activity assay after silencing of *MDM2* gene expression using siRNA (siMDM2#1, siMDM2#2). (**a**) Cell viability following downregulation of *MDM2* gene expression in a panel of breast cancer cell lines. The viability was assayed 72 h post-transfection and normalized to cells transfected with non-specific siRNA (control). (**b**) Viability of SKBR3 cells (HER2-enriched subtype) transfected with non-specific siRNA (control) or one of two different *MDM2*-silencing siRNAs (siMDM2#1, siMDM2#2) and treated for 72 h with various concentrations of DNA-damaging chemotherapeutic agents (camptothecin, doxorubicin, etoposide). For each transfection viability was normalized to non-treated cells. Statistical significance was assessed by comparing two silencing sequences (siMDM2#1, siMDM2#2) with the control (non-specific siRNA) at each concentration of the chemotherapeutic agent, * *p* < 0.05, ** *p* < 0.01, *** *p* < 0.001. Three independent biological experiments were performed for each chemotherapeutic (*n* = 3).

To elucidate the molecular mechanism of these clinical observations, we tested, using a metabolic survival assay, the effect of *MDM2*-gene-silencing (siMDM2#1, siMDM2#2) on a panel of breast cancer cell lines: MCF7 (luminal subtype, with wild-type *TP53*), SKBR3 (HER2-enriched subtype with mutated p53-R175H), MDA-MB-231 (basal subtype, with mutated p53-R280K), and MDA-MB-468 (basal subtype with mutated p53-R273H). As shown in Figure 2a, after silencing of the *MDM2* gene expression and in the absence of any additional treatment, a slight decrease in cell viability was observed only in MCF7 cells (*TP53* WT). This can probably be attributed to the activation of p53-dependent apoptosis resulting from the decrease in the MDM2 level. 

However, different survival characteristics were revealed when prior to survival assay, cells were treated with clinically used chemotherapeutics: doxorubicin (DOXO), etoposide (ETOP), and camptothecin (CPT), which introduce DNA damage (Figure 2b, and results not shown). In the case of SKBR3 cells (HER2-enriched subtype, p53-R175H), we observed the decrease in survival after drug treatment in cells with reduced expression of endogenous *MDM2*. Similar results were obtained using real-time growth monitoring using xCelligence technology (Figure S2).

### 3.3. Downregulation of MDM2 Gene Expression Inhibits H2AX Histone Phosphorylation after Treatment of SKBR3 Cells with DNA-Damaging Agents

**Figure 3:**
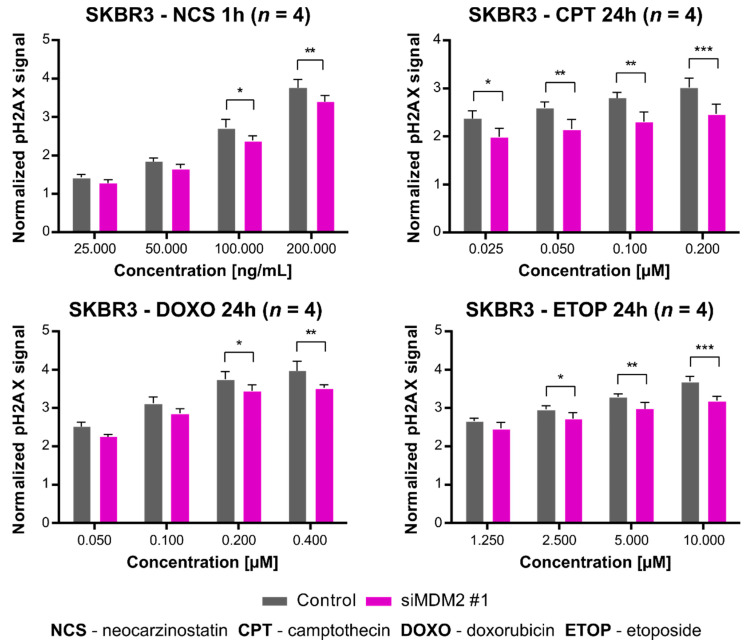
In-cell western analysis of histone H2AX phosphorylation after treatment with DNA-damaging agents and silencing of *MDM2* gene expression in the SKBR3 cells. The signal from H2AX phosphorylation was normalized to untreated cells. Statistical significance was assessed by comparing siMDM2 with the control (non-specific siRNA) for all tested concentrations of compounds, * *p* < 0.05, ** *p* < 0.01, *** *p* < 0.001. Four independent biological experiments were performed for each chemotherapeutic (*n* = 4).

To elucidate the molecular mechanism of this association, we used an in-cell western technique to assay phosphorylation of H2AX histone (γH2AX). Again, in the case of SKBR3 cells, we detected a correlation between the level of γH2AX and an siRNA-dependent decrease in *MDM2* expression, following treatment with NCS (neocarzinostatin), CPT, DOXO, and ETOP (Figure 3). Decreased γH2AX level following downregulation of *MDM2* expression could be explained by the role played by MDM2 in DNA damage repair (see the discussion in [45,47]). At the same time, we cannot exclude the possibility that the decrease in γH2AX levels after *MDM2* ablation with specific siRNA may also be partly due to enhanced apoptosis (see the results previously published in [39]). Both of these statements are consistent with the fact that in SKBR3 cells treated with chemotherapeutics, inhibition of *MDM2* expression reduced cell survival (Figure 2b).

### 3.4. Downregulation of MDM2 Gene Expression Reduces DNA Repair Efficiency by a Homologous Recombination Mechanism and Sensitizes SKBR3 Cells to Olaparib (Poly(ADP-Ribose) Polymerase, PARP, Inhibitor) 

**Figure 4:**
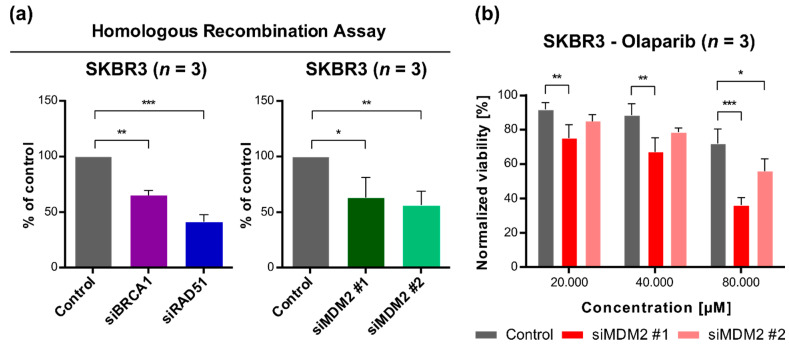
Analysis of homologous recombination DNA repair efficiency and sensitivity to olaparib in SKBR3 cells following *MDM2* gene silencing. (**a**) Homologous recombination assay assessment of the homologous recombination efficiency after silencing the expression of two genes important for the studied DNA repair pathway and *MDM2* gene (right panel). Statistical significance was assessed by comparing siRNA against the specific gene of interest with the control (non-specific siRNA). (**b**) Metabolic activity of SKBR3 cells (HER2-enriched subtype) treated for 72 h with olaparib (PARP inhibitor). The viability was normalized to untreated cells. Statistical significance was assessed by comparing two silencing sequences (siMDM2#1, siMDM2#1) with the control (non-specific siRNA) at each concentration of olaparib, * *p* < 0.05, ** *p* < 0.01, *** *p* < 0.001. *n*—the number of independent experiments performed.

To test whether MDM2 is involved in HR DNA repair we used the Homologous Recombination Assay Kit from Norgen Biotek. Components of the kit-plasmids containing various mutated lacZα cassettes that can form a functional lacZα cassette through intermolecular homologous recombination were transfected into SKBR3 cells. This assay, as employed here, is based on RT-PCR quantification of intact lacZα cassette re-created from the components of the kit by homologous recombination in cells. In the validation experiment, we tested whether the assay was sensitive to the presence of known factors directly involved in HR DNA repair, namely, BRCA1 and RAD51. The presence of specific siRNA against *BRCA1* or *RAD51* inhibited the recombination of transfected plasmids containing mutated lacZα cassettes when compared to the control (non-specific) siRNA (Figure 4a). Using the same assay, we showed that siRNAs against *MDM2* also impaired homologous recombination reaction, suggesting that MDM2 could be a positive factor required for HR DNA repair (Figure 4a). The positive involvement of MDM2 in HR-based DNA repair was also supported by the finding, that silencing of *MDM2* gene expression sensitized SKBR3 cells to PARP inhibitor, olaparib (Figure 4b). Olaparib is used in the clinic for the breast cancer patients with mutated *BRCA1* and/or *BRCA2* where the HR DNA repair is impaired [48]. 

### 3.5. MDM2 K454A Variant, Which Lacks Chaperone-like Activity and Inhibits Homologous Recombination DNA Repair in H1299 Non-Small-Cell Lung Cancer Cells 

The interpretation of the results obtained in SKBR3 cells, where MDM2 may act as positive factor for HR-based DNA repair, is difficult since these cells express mutated p53-R175H that also interacts with MDM2 [39] and MRN complexes [49]. We engineered the SKBR3 cell line with stably silenced p53 expression, effectively a (*TP53*^−/−^) variant, but the morphology of these cells differed significantly from the parental cell line. We decided that, since silencing of *TP53* apparently introduced profound changes in cellular metabolism, the results obtained with parental and modified cells would be difficult to compare. Therefore, we chose H1299 non-small-cell lung cancer (NSCLC) cells, which are *TP53*^−/−^. Moreover, the expression of endogenous MDM2 in H1299 cells is very low [39]. In these cells, exogenous MDM2 (both WT and mutated variants) was expressed by transfection with an appropriate plasmid construct. The validity of the new experimental model (cell line) was also supported by the observation that high expression of *MDM2* was associated with a poor prognosis for NSCLC cancer patients [28]. 

**Figure 5:**
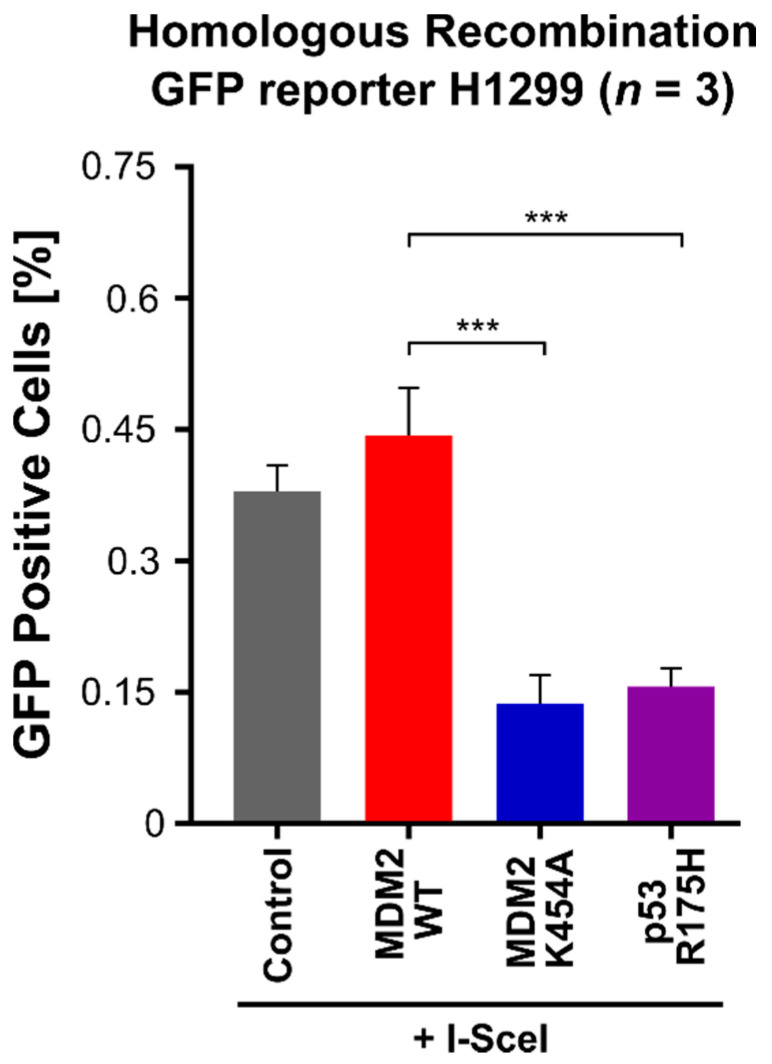
Analysis of homologous recombination DNA repair efficiency. Homologous recombination GFP reporter assessment after overexpression of MDM2 WT, MDM2 K454A, and p53 R175H. Statistical significance was assessed by comparing MDM2 WT overexpression with other samples, *** *p* < 0.001. *n*—the number of independent experiments performed.

In order to check if exogenous MDM2 is involved in homologous recombination in H1299 cells, we used the cells with stable expression of the GFP/GFP* HR reporter [50]. This construct, when cut by the I-SceI restriction enzyme and processed by cellular homologous recombination machinery, produces a functional GFP reading frame and green fluorescence in the cells. These reporter cells were transiently transfected with plasmids expressing MDM2 WT, MDM2 K454A (chaperone-dead variant), or p53 R175H. Twenty-four hours later, the cells were transfected with plasmid expressing I-SceI restriction enzyme in order to introduce double-strand breaks in the reporter construct. After an additional 48 h, the level of active GFP was measured. As shown in Figure 5, expression of the restriction enzyme I-SceI stimulated homologous recombination of GFP (control). Additional expression of exogenous MDM2 WT did not change the recombination of the GFP in a statistically significant manner. Interestingly, overexpression of mutated MDM2 K454A, which abolishes binding of ATP to MDM2 and inhibits its molecular chaperone-like activity [20], inhibited homologous recombination of *GFP* genes. Similar results were obtained when H1299 cells were transfected with mutated p53 R175H. This last result additionally supports previous findings that mutated p53 interacts and interferes with the MRN complex, therefore impairing HR-based DNA repair [49]. 

### 3.6. MDM2 WT Stimulates While MDM2 K454A Inhibits Phosphorylation of Multiple Proteins Involved in DDR (ATM, BRCA1, NBN) in H1299 Cells with DSBs

**Figure 6:**
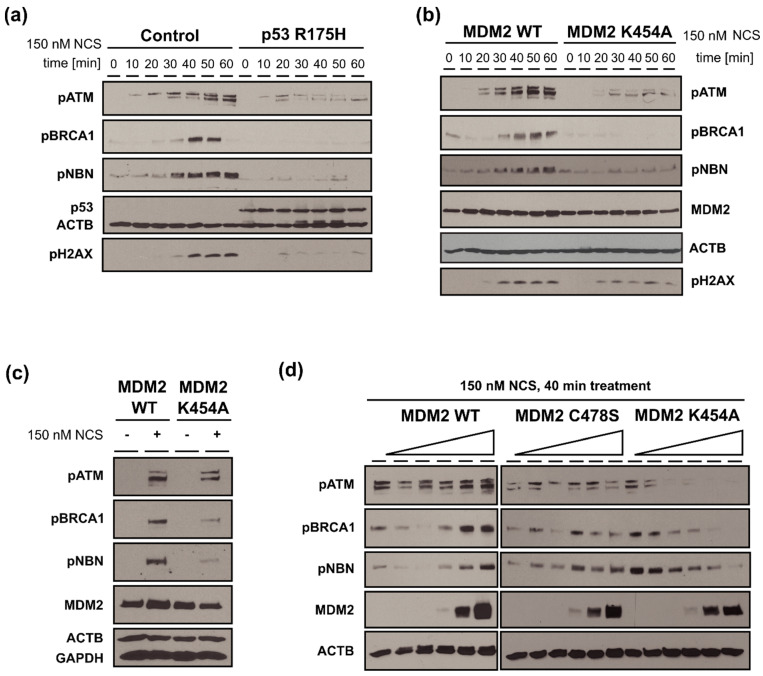
Analysis of the phosphorylation of proteins involved in DDR (ATM, BRCA1, NBN) after treatment with DNA-damaging agent in the H1299 cells overexpressing MDM2 variants. (**a**,**b**) Western blot analysis of NCS-treated cells with specific gene overexpression or empty vector (control). Time-course from 0 to 60 min after treatment with 150 nM of NCS. (**c**) Western blot analysis of MDM2 WT and K454A mutant at 40 min with and without 150 nM NCS treatment. (**d**) Western blot analysis of NCS-treated cells transfected with increasing amounts of expression vectors, at 40 min after 150 nM NCS treatment. Representative western blots shown.

The results of experiments showing the kinetics of phosphorylation of proteins involved in DSBs repair are presented in Figure 6a,b. Without additional expression of *MDM2*, phosphorylation of ATM kinase (pATM) after NCS treatment (time 0) was a very fast process and corresponded to the kinetics of NBN phosphorylation. Phosphorylation of BRCA1 (pBRCA1) was slightly delayed compared to the phosphorylation of ATM (pATM) and NBN (pNBN), suggesting that this event occurred after MRN-dependent activation of ATM (Figure 6a). Expression of mutant p53-R175H protein substantially inhibited the phosphorylation of ATM and almost completely abolished the phosphorylation of H2AX, NBN, and BRCA1 (Figure 6a). After ectopic expression of MDM2 WT, the phosphorylation of the above proteins was slightly accelerated/stimulated (Figure 6b). However, expression of the MDM2 K454A variant (after NCS treatment) substantially inhibited phosphorylation of ATM, BRCA1, NBN, and H2AX (Figure 6b). Based on the kinetics of phosphorylation of these proteins, the 40-min time-point was chosen to investigate the effect of overexpression of various variants of MDM2, namely: WT, chaperone dead (K454A), and E3-ligase dead (C478S) on the phosphorylation of proteins involved in DDR.

The activation of ATM (phosphorylation of serine S1981), BRCA1 (phosphorylation of serine S1524), and NBN (phosphorylation of serine S343) occurred only when both MDM2 WT and NCS were present (Figure 6c). Interestingly, we also observed MDM2-independent phosphorylation of those proteins, suggesting the existence of different mechanisms of DSBs DNA repair activation (see the discussion section for details). Substitution of MDM2 WT with the MDM2 K454A variant inhibited phosphorylation of these proteins in a dose-dependent manner. Phosphorylation of NBN, BRCA1, and, to some extent, ATM was stimulated by increasing expression of MDM2 WT (Figure 6d). Substitution of MDM2 WT with mutant MDM2 C478S with abolished E3-ligase activity did not impair the stimulation of NBN and BRCA1 phosphorylation, which suggests that E3-ligase activity of MDM2 was not required for activation of those proteins. It was shown before that other E3 ligases are involved in ubiquitination of NBN leading to activation of HR DNA repair reaction [51,52,53]. Interestingly, increasing expression of mutant MDM2 K454A, as well as double mutant MDM2 K454A C478S (result not shown), substantially inhibited phosphorylation of ATM, BRCA1, and NBN (Figure 6d). 

### 3.7. Chaperone-Dead MDM2 K454A Variant Interacts with NBN More Efficiently Than MDM2 WT

**Figure 7:**
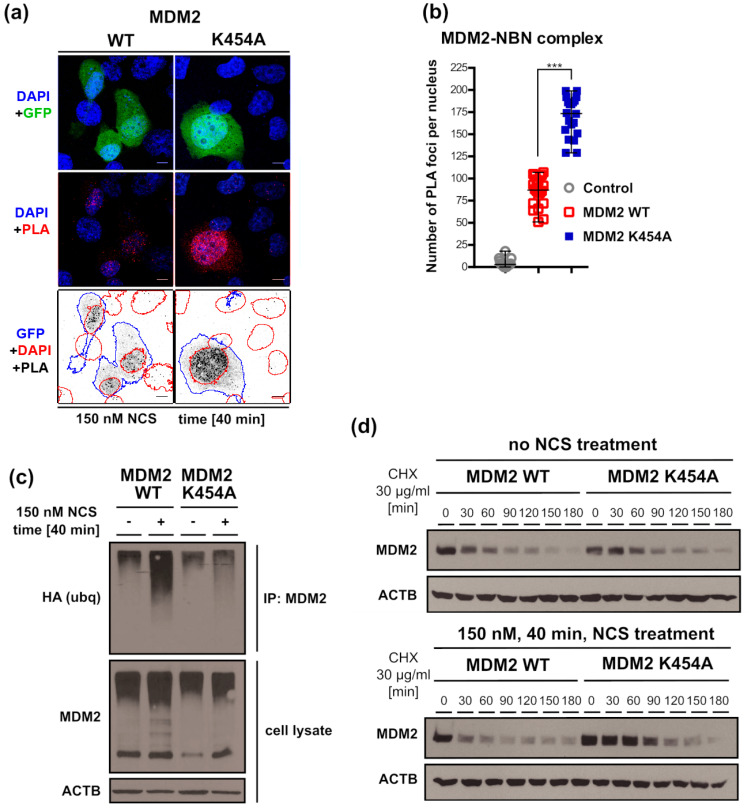
Analysis of NBN–MDM2 interaction efficiency and MDM2 stability after NCS treatment. (**a**) Proximity ligation assay between MDM2 and NBN, performed at 40 min after 150 nM NCS treatment. Representative fields of view shown for MDM2 WT and K454A transfections. GFP is a co-transfection marker for MDM2 expression. Red fluorescence (PLA) represents direct proximity of MDM2 and NBN. In the bottom panel, the PLA signal is represented by black dots, and the location of nuclei is marked by red outlines and GFP by blue outlines. GFP-positive cells are also MDM2-overexpressing cells. (**b**) Quantification of PLA foci number per nucleus. Statistical significance was assessed by comparing overexpression of MDM2 WT and K454A, *** *p* < 0.001. Control transfection with GFP plasmid. (**c**) In-vivo protein ubiquitination assay comparing MDM2 WT and K454A ubiquitination efficiency 40 min after treatment with 150 nM NCS. (**d**) Western blot analysis of protein stability assayed in the presence of translation inhibitor cycloheximide. Comparison of MDM2 WT to K454A variant with and without NCS treatment (150 nM, 40 min), over a 180 min time-course. Representative western blot shown.

Using a proximity ligation assay (PLA), we demonstrated that, in H1299 cells expressing exogenous MDM2 and treated with NCS for 40 min, NBN and MDM2 were located in direct proximity, suggesting that they interacted with each other in the nucleus (Figure 7a). Surprisingly, substitution of MDM2 WT with MDM2 K454A increased the proximity signal, suggesting that MDM2 K454A interacted with NBN more efficiently (Figure 7a,b). 

A possible interpretation of these results is that NCS treatment activated ATM, which phosphorylated both MDM2 and NBN and caused MDM2–NBN complex dissociation. In the presence of MDM2 K454A, phosphorylation of NBN was inhibited (Figure 6c,d), and mutated MDM2 K454A did not dissociate from the MRN complex (Figure 7b). Assuming that this interpretation is correct, the MDM2 WT dissociating from the MRN complex might be more prone to ubiquitination and thus more unstable than MDM2 K454A. Indeed, NCS treatment of the cells expressing MDM2 WT induces efficient ubiquitination of this protein, while the effect of NCS on MDM2 K454A ubiquitination is not so prominent (Figure 7c).

In support of this, we showed that, without NCS treatment, the degradation rates of MDM2 WT and MDM2 K454A were similar (Figure 7d). However, after NCS/cycloheximide treatment, MDM2 WT was degraded faster than the MDM2 K454A variant (Figure 7d). 

### 3.8. Overexpression of MDM2 WT Gene Led to Longer Proliferation of H1299 Cells (NSCLC) after Introduction of DNA Damage

**Figure 8:**
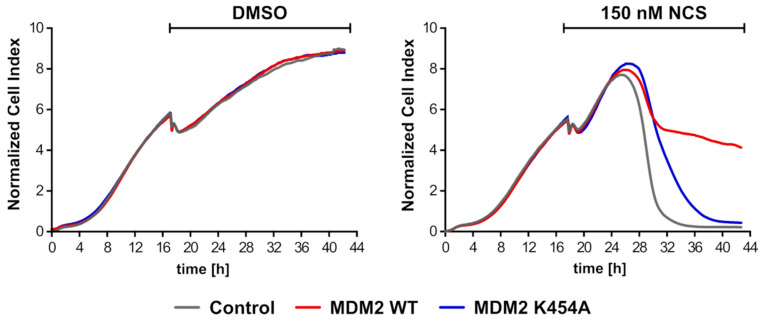
Acquisition of chemoresistant phenotype by MDM2-overexpressing H1299 cells. Real-time cell proliferation monitoring was performed with the RTCA DP xCelligence system; cells were transfected with MDM2 WT, MDM2 K454A or GFP plasmid (Control) and treated with 150 nM NCS or DMSO as the treatment control.

Our results suggest that once liberated from MDM2, the MRN complex located on DSB activates ATM and allows the HR-based DNA repair reaction to proceed. Efficient repair of DNA DSB can inhibit drug-induced apoptosis and lead to the acquisition of chemoresistance by cancer cells. To test this scenario, we monitored H1299 cells proliferation using xCelligence technique (Figure 8). We observed that, 8 h after NCS treatment, the proliferation of cells was inhibited. The overexpression of MDM2 WT allowed for longer proliferation of the cells, suggesting the acquisition of partial resistance to NCS treatment. The overexpression of mutant MDM2 K454A delayed the response to the drug, but, 22 h after NCS treatment, the proliferation of H1299 cells was completely inhibited. The molecular mechanism of chemoresistance acquisition resulting from high expression of MDM2, which we proposed here, might contribute to the poor prognosis for breast cancer (this article, Figure 1) and non-small cell lung cancer [28] patients with high expression of MDM2.

## 4. Discussion

Amplification of *MDM2* or *MDM4* genes in some cancers results in decreased patient survival [54]. We showed that *MDM2* gene amplification is a factor independent of the p53 status in the prognosis of non-small-cell lung cancer [28]. It was demonstrated, using immunostaining, that MDM2 overexpression as well as its nuclear localization are negative prognostic markers in breast carcinomas [40,55]. In this study, we showed that overexpression of the *MDM2* gene clearly correlates with decreased overall survival of breast cancer patients, but only in the case of the HER2 molecular cancer subtype. A decreasing endogenous MDM2 level (using specific siRNA) in different breast cancer cell lines led to the same conclusions: only in the case of SKBR3 cells (which represent the HER2 subtype of breast cancer) did downregulation of *MDM2* gene result in the decrease in cell survival. Interestingly, *MDM2* gene silencing in SKBR3 cells diminished cell survival and proliferation after treatment with drugs introducing DNA damage (CPT, DOXO, ETOP), suggesting that the presence of the MDM2 protein could augment repair of DSB created by these drugs. In support of this notion, the decrease in *MDM2* expression reduced phosphorylation of H2AX protein involved in the repair of DSBs and inhibited homologous recombination, suggesting that MDM2 could be a positive factor involved in the HR-based DNA repair process.

The interpretation of these results is difficult because SKBR3 cells contain mutated *TP53* (p53 R175H variant), which could itself, by binding to MRN complex, influence the repair of DSBs [49]. Moreover, it was shown before that, when p53 is not functional, inhibition of MDM2 could induce expression of the TAp73α tumor suppressor protein and promote p73-dependent apoptosis [47]. To complicate this picture even more, we showed that in SKBR3 cells the endogenous level of MDM2 was sufficient for the formation of a p53 R175H-TAp73α-MDM2 complex, which inhibited TAp73α-dependent apoptosis of SKBR3 cancer cells [39]. In that scenario, MDM2 worked as a scaffold protein, stabilizing the interaction between p53 R175H and TAp73α. To distinguish the effect of mutated p53 R175H protein from the effect of overexpression of the *MDM2* gene on the repair of DSBs, we tried to engineer a genetically stable SKBR3 (*TP53*^−/−^), which proved unfeasible. Therefore, we employed a simpler system: the H1299 (*TP53*^−/−^) cell line, which also expressed a very low level of endogenous MDM2. Increasing *MDM2* expression led to an increase in the phosphorylation of NBN, BRCA1, and, to some extent, ATM. In the case of γH2AX, however, increased expression of MDM2 partially inhibited and delayed the appearance of phosphorylated H2AX histone. These findings support the idea that, initially, before activation of ATM, phosphorylation of H2AX near DSB is conducted by VRK1 chromatin protein kinase [56]. After activation of ATM, the ATM-dependent phosphorylation of H2AX helps to assemble and stabilize protein complexes involved in DNA repair [57,58].

We also demonstrated that overexpression of the MDM2 K454A variant (instead of MDM2 WT) diminishes phosphorylation of ATM, NBN, and BRCA1 and weakens phosphorylation of H2AX histone. We had previously shown that MDM2 WT possessed ATP-dependent molecular chaperone-like activity since it could substitute HSP90 in the folding of p53 and luciferase proteins in an ATP-dependent reaction [20]. MDM2 WT bound ATP but did not catalyze its hydrolysis. The K454A mutation abrogated binding of ATP to MDM2 and blocked its chaperone-like activity, but it did not block its E3-ligase activity [26]. As we demonstrated here, the affinity of MDM2 K454A towards NBN, in cells treated with NCS, was higher than that of MDM2 WT, suggesting that after drug treatment MDM2 WT dissociates from NBN, which is a component of the MRN complex located on DSB. This scenario is supported by the finding that, after NCS treatment, MDM2 WT was more efficiently ubiquitinated and degraded by proteasome than MDM2 K454A. We addressed the question whether MDM2 WT was involved in ATM activation by the MRN complex located on the DSB and why the chaperone-like activity of MDM2 WT was important in this process.

One possible scenario is that MDM2 helps to bring non-active ATM to the MRN complex. Then, following activation of ATM and ATM-dependent phosphorylation of MDM2, the latter dissociates from MRN complex, thus allowing ATM-dependent phosphorylation of proteins involved in HR-based DNA repair. It was shown before that non-active ATM kinase was attracted to the MRN complex positioned on DSB. The carboxy-terminus of NBN was shown to directly interact with ATM, contributing to its activation [58,59,60,61]. It cannot be ruled out that the NBN-MDM2 complex augments the loading of ATM on the MRN complex positioned on DSB. Recently, it was shown that HSP90α-ATM and HSP90α-NBN complexes exist in unstressed (non-irradiated) cells, contributing to the ATM and NBN stability that is required for the MRN-complex-dependent ATM activation. HSP90 helps to load ATM on the MRN complex; after ATM activation, it phosphorylates HSP90, thus triggering its dissociation from the MRN-ATM complex [62,63]. Interestingly, the presence of HSP90 inhibitor, 17-DMAG, reduced the interaction between NBN and ATM, radiation-induced activation of ATM, and the ability of MRN components to form nuclear foci after irradiation [64]. Given that MDM2, due to its chaperone-like activity, can replace HSP90 in promoting p53 binding to its corresponding promoter sequence [20], the case may also be true for ATM interactions. As shown before, NBN interacted with MDM2; even NBN mutant lacking both the N- and C-terminus still co-immunoprecipitated with MDM2 [43]. More detailed analysis demonstrated that the region of NBN comprising 474aa to 512aa is responsible for the interaction [44]. Notably, MDM2 can also interact with ATM [65]. These combined findings suggest that MDM2 might work as a scaffolding protein, interacting with both ATM and NBN, helping to position the former near the C-terminus of the latter. At the same time, the prolonged presence of MDM2 interferes with the activation of ATM; therefore, MDM2 must be released from the complex, allowing for the proper interaction of its components. Activated ATM phosphorylates MDM2, ensuring its autoubiquitination and proteasomal degradation [66].

The alternative scenario is that MDM2 simply acts as an inhibitor that needs to dissociate from the MRN complex to trigger the activation of ATM. Another molecular chaperone, such as the previously mentioned HSP90, could by itself be responsible for guiding ATM to the MRN complex. This scenario is supported by the finding that, in our homologous recombination-based GFP assay, after creation of DSB by expression of I-SceI restriction enzyme, in the presence of low concentration of endogenous MDM2, GFP recombination still occurred. An increasing concentration of endogenous MDM2 improved this reaction only slightly, and overexpression of MDM2 did not inhibit recombination. The only thing that could significantly inhibit GFP recombination was substitution of MDM2 WT by MDM2 K454A. A similar effect was observed when phosphorylation of NBN or BRCA1 was investigated—an increase in MDM2 expression caused an increase in pNBN and pBRCA1 levels in a dose-dependent manner. 

Our results demonstrate that MDM2 needs to dissociate from NBN to enable activation of ATM protein kinase, which is associated with MRN positioned on DSB. Even a single point mutation in the ATP-binding site of MDM2 (K454A), which diminishes dissociation of MDM2 from the complex with NBN, could block the activation of ATM. As shown previously by the Eischen laboratory and others (review in [25]), MDM2 and MDM4 bind to MRN complex by specific interaction with NBN and inhibit the repair of DSB. Thus, inhibition of DNA repair by MDM2 helps cells survive the presence of DSBs by increasing genomic instability, which leads to cellular transformation [25]. In transformed cancer cells, particularly those without functional p53, the selective pressure is probably directed towards the overproduction of MDM2. This is mainly because of increased survival of those cells (MDM2 abrogates activity of other tumor-suppressor proteins like Rb and E2F1). 

## 5. Conclusions

**Figure 9:**
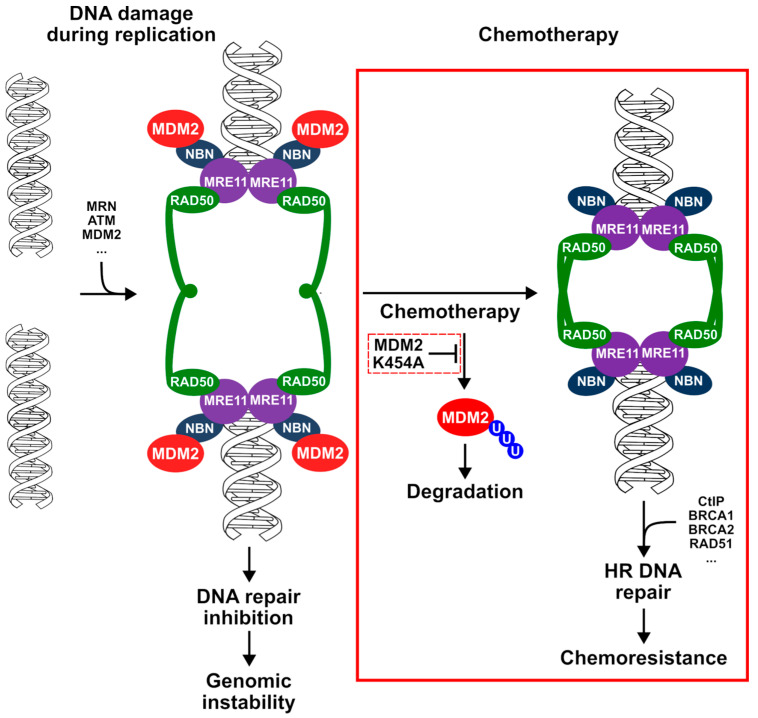
Proposed model of MDM2 protein action during DNA double-strand breaks repair after treatment with DNA-damaging chemotherapeutics. Chemotherapy-induced double-strand breaks stimulate activation of the kinases responsible for MDM2 dissociation from the MRN complex; MDM2 is ubiquitinated and degraded, opening the way for effective DNA repair through the homologous recombination mechanism and resulting in the acquisition of chemoresistance. The novel elements of the model proposed on the basis of the results presented in our study are marked with a red box.

Previous studies in cancer cells not treated with chemotherapeutics have shown that the interaction between MDM2 and NBN inhibits the repair of DSBs generated during DNA replication in rapidly proliferating cells. The data presented here demonstrate that the situation is radically different when cancer cells are treated with chemotherapy (Figure 9). Such treatment efficiently activates stress kinases ATM and CHK2, which, in turn, phosphorylate MDM2. This phosphorylation causes dissociation of MDM2 from the MRN complex located on DSB and triggers the initiation of HR-based DNA repair. Unfortunately, the increase in DSB repair efficiency in cancer cells with high MDM2 expression translates into clinical acquisition of a chemoresistant phenotype.

## Data Availability

The data that support the findings of this study are available on reasonable request from the corresponding authors.

## Supplementary Materials

The following are available online at https://www.mdpi.com/article/10.3390/cancers13184501/s1, Figure S1: TCGA Breast Cancer (BRCA) dataset details, Figure S2: Real-time monitoring of SKBR3 cells growth after MDM2 gene silencing and treatment.

## References

[1] Desai A., Yan Y., Gerson S.L. (2018). Advances in therapeutic targeting of the DNA damage response in cancer. DNA Repair.

[2] Lavin M.F., Kozlov S., Gatei M., Kijas A.W. (2015). ATM-Dependent Phosphorylation of All Three Members of the MRN Complex: From Sensor to Adaptor. Biomolecules.

[3] Chernikova S., Game J.C., Brown J.M. (2012). Inhibiting homologous recombination for cancer therapy. Cancer Biol. Ther..

[4] Sakamoto Y., Kokuta T., Teshigahara A., Iijima K., Kitao H., Takata M., Tauchi H. (2021). Mitotic cells can repair DNA double-strand breakes via a homology-directed pathway. J. Rad. Res..

[5] Casari E., Rinaldi C., Marsella A., Gnugnoli M., Colombo C.V., Bonetti D., Longhese M.P. (2019). Processing of DNA double-strand breaks by the mrx complex in a chromatin context. Front. Mol. Biosci..

[6] Shibata A., Jeggo P., Lobrich M. (2018). The pendulum of the ku-ku clock. DNA Repair.

[7] Ströbel T., Madlener S., Tuna S., Vose S., Lagerweij T., Wurdinger T., Vierlinger K., Wöhrer A., Price B.D., Demple B. (2017). Ape1 guides DNA repair pathway choice that is associated with drug tolerance in glioblastoma. Sci. Rep..

[8] Wen J., Cerosaletti K., Schultz K.J., Wright J.A., Concannon P. (2013). NBN Phosphorylation regulates the accumulation of MRN and ATM at sites of DNA double-strand breaks. Oncogene.

[9] Sullivan M.R., Bernstein K.A. (2018). RAD-ical New Insights into RAD51 Regulation. Genes.

[10] Maki C.G., Huibregtse J.M., Howley P. (1996). In vivo ubiquitination and proteasome-mediated degradation of p53(1). Cancer Res..

[11] Vogelstein B., Lane D., Levine A.J. (2000). Surfing the p53 network. Nature.

[12] Haupt S., Buckley D., Pang J.-M., Panimaya J., Paul P.J., Gamell C., Takano E., Lee Y.Y., Hiddingh S., Rogers T.-M. (2015). Targeting Mdmx to treat breast cancers with wild-type p53. Cell Death Dis..

[13] Haupt S., Vijayakumaran R., Miranda P.J., Burgess A., Lim E., Haupt Y. (2017). The role of mdm2 and mdm4 in breast can-cer development and prevention. J. Mol. Cell Biol..

[14] Manfredi J.J. (2010). The Mdm2-p53 relationship evolves: Mdm2 swings both ways as an oncogene and a tumor suppressor. Genes Dev..

[15] Jacob A.G., Singh R.K., Comiskey D.F., Rouhier M.F., Mohammad F., Bebee T.W., Chandler D.S. (2014). Stress-Induced Alternative Splice Forms of MDM2 and MDMX Modulate the p53-Pathway in Distinct Ways. PLoS ONE.

[16] Bieging K.T., Mello S.S., Attardi L.D. (2014). Unravelling mechanisms of p53-mediated tumour suppression. Nat. Rev. Cancer.

[17] Hollstein M., Sidransky D., Vogelstein B., Harris C.C. (1991). p53 mutations in human cancers. Science.

[18] Oren M., Rotter V. (2010). Mutant p53 Gain-of-Function in Cancer. Cold Spring Harb. Perspect. Biol..

[19] Honda R., Tanaka H., Yasuda H. (1997). Oncoprotein MDM2 is a ubiquitin ligase E3 for tumor suppressor p53. FEBS Lett..

[20] Wawrzynow B., Zylicz A., Wallace M., Hupp T., Zylicz M. (2007). MDM2 Chaperones the p53 Tumor Suppressor. J. Biol. Chem..

[21] Oliner J.D., Pietenpol J.A., Thiagalingam S., Gyuris J., Kinzler K.W., Vogelstein B. (1993). Oncoprotein mdm2 conceals the ac-tivation domain of tumour suppressor p53. Nature.

[22] Momand J., Zambetti G.P., Olson D.C., George D., Levine A.J. (1992). The mdm-2 oncogene product forms a complex with the p53 protein and inhibits p53-mediated transactivation. Cell.

[23] Malbert-Colas L., Ponnuswamy A., Olivares-Illana V., Tournillon A.-S., Naski N., Fåhraeus R. (2014). HDMX Folds the Nascent p53 mRNA following Activation by the ATM Kinase. Mol. Cell.

[24] Bohlman S., Manfredi J.J. (2016). Mdm2-RNA Interactions as a Target for Cancer Therapy: It’s Not All About p53. Cancer Cell.

[25] Eischen C.M. (2017). Role of Mdm2 and Mdmx in DNA repair. J. Mol. Cell Biol..

[26] Bohlman S., Manfredi J.J. (2014). p53-Independent Effects of Mdm2. Subcell Biochem..

[27] Cordon-Cardo C., Latres E., Drobnjak M., Oliva M.R., Pollack D., Woodruff J.M., Marechal V., Chen J., Brennan M.F., Levine A.J. (1994). Molecular abnormalities of mdm2 and p53 genes in adult soft tissue sarcomas. Cancer Res..

[28] Dworakowska D., Jassem E., Jassem J., Peters B., Dziadziuszko R., Zylicz M., Jakobkiewicz-Banecka J., Kobierska-Gulida G., Szymanowska A., Skokowski J. (2004). Mdm2 gene amplification: A new independent factor of ad-verse prognosis in non-small cell lung cancer (nsclc). Lung Cancer.

[29] Sheikh M.S., Shao Z.M., Hussain A., Fontana J. (1993). The p53-binding protein *MDM2* gene is differentially expressed in human breast carcinoma. Cancer Res..

[30] Watanabe T., Ichikawa A., Saito H., Hotta T. (1996). Overexpression of the *MDM2* Oncogene in Leukemia and Lymphoma. Leuk. Lymphoma.

[31] Momand J., Jung D., Wilczynski S., Niland J. (1998). The *MDM2* gene amplification database. Nucleic Acids Res..

[32] Onel K., Cordon-Cardo C. (2004). MDM2 and prognosis. Mol. Cancer Res..

[33] Bond G.L., Hu W., Bond E.E., Robins H., Lutzker S.G., Arva N.C., Bargonetti J., Bartel F., Taubert H., Wuerl P. (2004). A single nucleotide polymorphism in the MDM2 promoter attenuates the p53 tumor suppressor pathway and acceler-ates tumor formation in humans. Cell.

[34] Rayburn E., Zhang R., He J., Wang H. (2005). MDM2 and Human Malignancies: Expression, Clinical Pathology, Prognostic Markers, and Implications for Chemotherapy. Curr. Cancer Drug Targets.

[35] Bond G.L., Hirshfield K.M., Kirchhoff T., Alexe G., Bond E.E., Robins H., Bartel F., Taubert H., Wuerl P., Hait W. (2006). MDM2 SNP309 Accelerates Tumor Formation in a Gender-Specific and Hormone-Dependent Manner. Cancer Res..

[36] Brekman A., Singh K., Polotskaia A., Kundu N., Bargonetti J. (2011). A p53-independent role of MDM2 in estrogen-mediated activation of breast cancer cell proliferation. Breast Cancer Res..

[37] Deb S.P., Singh S., Deb S. (2014). MDM2 overexpression, activation of signaling networks, and cell proliferation. Subcell Biochem..

[38] Yu Q., Li Y., Mu K., Li Z., Meng Q., Wu X., Wang Y., Li L. (2014). Amplification of Mdmx and overexpression of MDM2 contribute to mammary carcinogenesis by substituting for p53 mutations. Diagn. Pathol..

[39] Tracz-Gaszewska Z., Klimczak M., Biecek P., Herok M., Kosinski M., Olszewski M., Czerwińska P., Wiech M., Wiznerowicz M., Zylicz A. (2017). Molecular chaperones in the acquisition of cancer cell chemoresistance with mutated *TP53* and MDM2 up-regulation. Oncotarget.

[40] Park H.S., Park J.M., Park S., Cho J., Kim S.I., Park B.-W. (2014). Subcellular localization of Mdm2 expression and prognosis of breast cancer. Int. J. Clin. Oncol..

[41] Hernández-Monge J., Rousset-Roman A.B., Medina-Medina I., Olivares-Illana V. (2016). Dual function of MDM2 and MDMX toward the tumor suppressors p53 and RB. Genes Cancer.

[42] Stevens C., Pettersson S., Wawrzynow B., Wallace M., Ball K., Zylicz A., Hupp T.R. (2008). ATP stimulates MDM2-mediated inhibition of the DNA-binding function of E2F1. FEBS J..

[43] Alt J.R., Bouska A., Fernandez M., Cerny R.L., Xiao H., Eischen C.M. (2005). Mdm2 Binds to Nbs1 at Sites of DNA Damage and Regulates Double Strand Break Repair. J. Biol. Chem..

[44] Bouska A., Lushnikova T., Plaza S., Eischen C.M. (2008). Mdm2 Promotes Genetic Instability and Transformation Independent of p53. Mol. Cell. Biol..

[45] Senturk J.C., Bohlman S., Manfredi J.J. (2017). Mdm2 selectively suppresses DNA damage arising from inhibition of topoiso-merase ii independent of p53. Oncogene.

[46] Conradt L., Henrich A., Wirth M., Reichert M., Lesina M., Algül H., Schmid R.M., Krämer O.H., Saur D., Schneider G. (2013). Mdm2 inhibitors synergize with topoisomerase II inhibitors to induce p53-independent pancreatic cancer cell death. Int. J. Cancer.

[47] Feeley K.P., Adams C.M., Mitra R., Eischen C.M. (2017). Mdm2 Is Required for Survival and Growth of p53-Deficient Cancer Cells. Cancer Res..

[48] Lyons T.G., Robson M.E. (2018). Resurrection of PARP Inhibitors in Breast Cancer. J. Natl. Compr. Cancer Netw..

[49] Liu D.-P., Song H., Xu Y. (2010). A common gain of function of p53 cancer mutants in inducing genetic instability. Oncogene.

[50] Słabicki M., Theis M., Krastev D.B., Samsonov S., Mundwiller E., Junqueira M., Paszkowski-Rogacz M., Teyra J., Heninger A.-K., Poser I. (2010). A Genome-Scale DNA Repair RNAi Screen Identifies SPG48 as a Novel Gene Associated with Hereditary Spastic Paraplegia. PLoS Biol..

[51] Lu C.S., Truong L.N., Aslanian A., Shi L.Z., Li Y., Hwang P.Y., Koh K.H., Hunter T., Yates J.R., Berns M.W. (2012). The ring finger protein rnf8 ubiquitinates nbs1 to promote DNA double-strand break repair by homologous recombination. J. Biol. Chem..

[52] Wu J., Zhang X., Zhang L., Wu C.Y., Rezaeian A.H., Chan C.H., Li J.M., Wang J., Gao Y., Han F. (2012). Skp2 e3 lig-ase integrates atm activation and homologous recombination repair by ubiquitinating nbs1. Mol. Cell.

[53] Ha G.H., Ji J.H., Chae S., Park J., Kim S., Lee J.K., Kim Y., Min S., Park J.M., Kang T.H. (2019). Pellino1 regulates re-versible atm activation via nbs1 ubiquitination at DNA double-strand breaks. Nat. Commun..

[54] Eischen C.M., Lozano G. (2014). The Mdm network and its regulation of p53 activities: A rheostat of cancer risk. Hum. Mutat..

[55] Turbin D., Cheang M.C.U., Bajdik C.D., Gelmon K., Yorida E., De Luca A., Nielsen T., Huntsman D.G., Gilks C.B. (2006). MDM2 protein expression is a negative prognostic marker in breast carcinoma. Mod. Pathol..

[56] Campillo-Marcos I., Lazo P.A. (2018). Implication of the VRK1 chromatin kinase in the signaling responses to DNA damage: A therapeutic target?. Cell. Mol. Life Sci..

[57] Scully R., Xie A. (2013). Double strand break repair functions of histone H2AX. Mutat. Res. Mol. Mech. Mutagen..

[58] Shiloh Y. (2006). The ATM-mediated DNA-damage response: Taking shape. Trends Biochem. Sci..

[59] Lee J.H., Paull T.T. (2004). Direct activation of the atm protein kinase by the mre11/rad50/nbs1 complex. Science.

[60] You Z., Chahwan C., Bailis J., Hunter T., Russell P. (2005). ATM Activation and Its Recruitment to Damaged DNA Require Binding to the C Terminus of Nbs1. Mol. Cell. Biol..

[61] Falck J., Coates J., Jackson S.P. (2005). Conserved modes of recruitment of ATM, ATR and DNA-PKcs to sites of DNA damage. Nature.

[62] Pennisi R., Antoccia A., Leone S., Ascenzi P., Di Masi A. (2017). Hsp90α regulates ATM and NBN functions in sensing and repair of DNA double-strand breaks. FEBS J..

[63] Stracker T.H. (2017). Chaperoning the DNA damage response. FEBS J..

[64] Dote H., Burgan W.E., Camphausen K., Tofilon P.J. (2006). Inhibition of hsp90 compromises the DNA damage response to radiation. Cancer Res..

[65] De Toledo S.M., Azzam E.I., Dahlberg W.K., Gooding T.B., Little J.B. (2000). Atm complexes with hdm2 and promotes its rap-id phosphorylation in a p53-independent manner in normal and tumor human cells exposed to ionizing radiation. Oncogene.

[66] Inuzuka H., Fukushima H., Shaik S., Wei W. (2010). Novel insights into the molecular mechanisms governing mdm2 ubiquiti-nation and destruction. Oncotarget.

