# Supplementary material for: Chemotherapy of HER2- and MDM2-Enriched Breast Cancer Subtypes Induces Homologous Recombination DNA Repair and Chemoresistance
Source: Cancers (Basel). 2021 Sep 7;13(18):4501. doi: 10.3390/cancers13184501 (PMC8471926; doi:10.3390/cancers13184501)
Supplement: Supplementary file 1 [file cancers-13-04501-s001.zip › cancers-1342627-supplementary/cancers-1342627-supplementary.pdf]

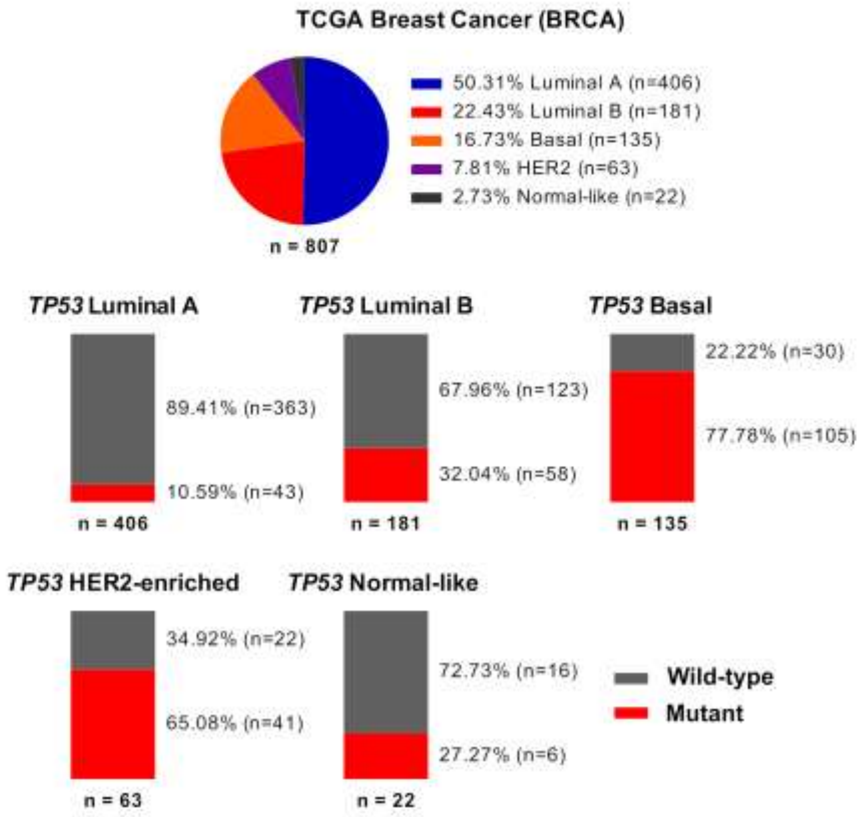

Figure S1. TCGA Breast Cancer (BRCA) dataset details. Percentage of different molecular subtypes of breast cancer in the dataset (top panel). Percentage of wild-type and mutant *TP53* in the specfic subtype (bottom panel).

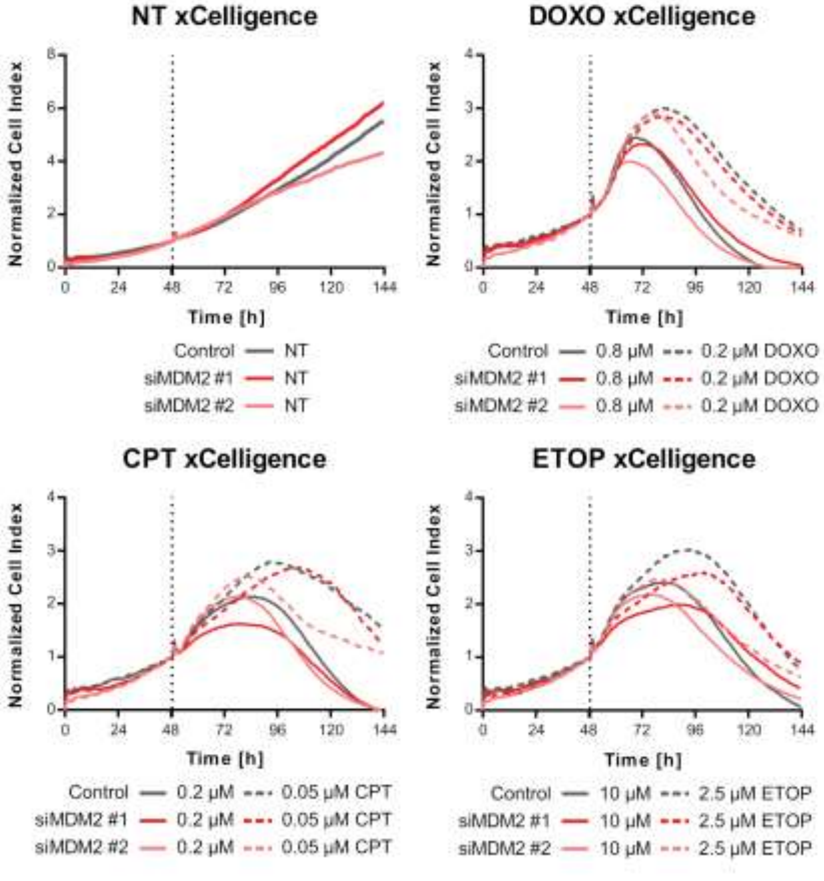

Figure S2. Real-time monitoring of SKBR3 cells growth after MDM2 gene silencing and treatment. Real-time proliferation monitoring was performed with the xCelligence System, SKBR3 cells were first transfected with specific siRNA and then treated with the indicated chemioterapeutic agent. Control indicates transfection with non-specific siRNA.
